# SeqDeχ: A Sequence Deconvolution Tool for Genome Separation of Endosymbionts From Mixed Sequencing Samples

**DOI:** 10.3389/fgene.2019.00853

**Published:** 2019-09-19

**Authors:** Alice Chiodi, Francesco Comandatore, Davide Sassera, Giulio Petroni, Claudio Bandi, Matteo Brilli

**Affiliations:** ^1^Department of Earth and Environmental Sciences, University of Pavia, Pavia, Italy; ^2^Department of Biosciences, University of Milan, Milan, Italy; ^3^Pediatric Clinical Research Center “Romeo ed Enrica Invernizzi”, University of Milan, Milan, Italy; ^4^Department of Biology and Biotechnology, University of Pavia, Pavia, Italy; ^5^Department of Biology, University of Pisa, Pisa, Italy; ^6^Department of Biomedical and Clinical Sciences “L. Sacco”, University of Milan, Milan, Italy

**Keywords:** symbiont, deconvolution, machine learning, binning, NGS

## Abstract

In recent years, the advent of NGS technology has made genome sequencing much cheaper than in the past; the high parallelization capability and the possibility to sequence more than one organism at once have opened the door to processing whole symbiotic consortia. However, this approach needs the development of specific bioinformatics tools able to analyze these data. In this work, we describe SeqDex, a tool that starts from a preliminary assembly obtained from sequencing a mixture of DNA from different organisms, to identify the contigs coming from one organism of interest. SeqDex is a fully automated machine learning–based tool exploiting partial taxonomic affiliations and compositional analysis to predict the taxonomic affiliations of contigs in an assembly. In literature, there are few methods able to deconvolve host–symbiont datasets, and most of them heavily rely on user curation and are therefore time consuming. The problem has strong similarities with metagenomic studies, where mixed samples are sequenced and the bioinformatics challenge is trying to separate contigs on the basis of their source organism; however, in symbiotic systems, additional information can be exploited to improve the output. To assess the ability of SeqDex to deconvolve host–symbiont datasets, we compared it to state-of-the-art methods for metagenomic binning and for host–symbiont deconvolution on three study cases. The results point out the good performances of the presented tool that, in addition to the ease of use and customization potential, make SeqDex a useful tool for rapid identification of endosymbiont sequences.

## Introduction

In recent years, we experienced a huge improvement in sequencing technologies. In particular, NGS machines have reached throughput levels and costs that make whole genome and metagenome sequencing technically easy and cheap.

In this article, we deal with a specific problem that arises when the sequencing is performed on heterogeneous DNA mixtures containing the DNA of a host and of its symbiont(s). Such “mixed samples” sequencing approach is widely used in the study of symbionts (Brown et al., 2016; Brown et al., 2018; Floriano et al., 2018), as growing these bacteria, particularly the intracellular ones, can be an extremely difficult and cumbersome endeavor.

In such cases, the comparative genomic analysis on both the host and the symbiont requires the sequences from either organism be identified, a procedure also called binning. One of the tools for binning sequences in mixed samples is called Blobology (Kumar et al., 2013), and it exploits two features that are specific to symbiotic associations involving prokaryotes and eukaryotes: first, the approach assumes that the symbiont has a skewed genomic composition with respect to the host, as observed for most symbiont genomes (Andersson and Kurland, 1998; Moran and Wernegreen, 2000; Gil et al., 2002; McCutcheon and Moran, 2012). Second, in the symbiotic systems studied so far, there are multiple symbiont cells per host’s cell, and this, together with the fact that prokaryotes often contain several genome copies, translates in a higher sequencing coverage (hereafter coverage) of DNA fragments from the symbiont genome. These two features are, however, empirical, and it is not possible to know if they hold true for different symbionts, especially in cases where the symbiosis is not obligatory or relatively young, such that the symbiont’s genomic properties have not completely adapted to the highly stable intracellular environment. The Blobology method starts with the alignment of the contigs to be classified against an appropriate sequence database to associate taxonomical categories to the contigs. The GC content and the coverage are used as a coordinate system for contig positioning in a 2D plot, and the taxonomy from the previous step is used as a color scheme. At this point, the user leverages the positions of the taxonomy-identified contigs to define a region that mostly contains sequences from the symbiont. As the partition cannot be perfect, a postprocessing of the results is necessary to reduce false-positives and false-negatives, usually by performing additional comparisons with sequence databases, eventually also at the protein level. This step is, however, time consuming, and the whole procedure highly subjective, as it requires the user to take important decisions on the basis of relatively few taxonomy informed points. As a consequence, there are no easy ways to assess how changing the region for selection affects the performances of the classification. When Blobology’s features do not allow to efficiently separate sequences from the host and the symbiont, most authors select contigs of interest based upon taxonomic affiliations obtained through comparison with public databases or databases specifically built with genomes of organisms related to those in the sample, or they exploit additional features, such as coverage, but this makes the procedure highly subjective (Brown et al., 2016; Kostygov et al., 2016; Small et al., 2016; Brown et al., 2018). Some authors perform additional steps to further reduce nontarget sequences. In some works, reads are mapped back to the assembled sequences, and only those mapping on target contigs are selected for reassembly; this is repeated iteratively until no more contigs are added or no more sequences are elongated (Chung et al., 2017). In other works, Blobology is performed on contigs selected on the basis of the expected taxonomic affiliations (Wang and Chandler, 2016), or sequences are manually inspected to try to locate overlapping sequences and obtain a circular bacterial chromosome (Kostygov et al., 2016). As in classical genome sequencing efforts, the use of different technologies has also been exploited to optimize the genome reconstructions, but this clearly requires a larger budget and high-quality DNA (Campbell et al., 2015; Husnik and McCutcheon, 2016; Floriano et al., 2018; Nikoh et al., 2018).

The problem of identifying DNA sequences of a symbiont in a sample that also contains host DNA bears strong similarities to the taxonomical binning used in metagenomics. However, the much higher complexity of DNA mixtures characteristic of metagenomic samples with respect to symbiotic systems make so that the algorithmic requirements are slightly different in the two cases. At the same time, we can make additional assumptions concerning the symbiotic systems that do not necessarily hold true for metagenomes. Therefore, existing metagenomic solutions could be appropriate with endosymbiotic systems, but specific tools could benefit by leveraging additional endosymbionts’ features. Several metagenomic tools address the problem of binning in metagenomic samples, and they can be (i) reference based (Seah and Gruber-Vodicka, 2015; Gregor et al., 2016) or (ii) reference-free (Teeling et al., 2004; Wu et al., 2014; Kang et al., 2019). Tools belonging to the former category obtain taxonomic annotation of contigs through homology searches, and the performances are consequently strongly dependent on the presence of related genome sequences in the reference database. As it has been recently demonstrated, this is rarely the case in metagenomic samples, especially for the less studied environments (Pasolli et al., 2019). Reference-free methods instead provide composition-based binning of the contigs, leveraging on the observation that DNA higher-order composition (based on k-mers of increasing length) provides a phylogenetic signal that is strongly correlated to alignment-based phylogenetic trees (Burge et al., 1992; Karlin et al., 1994). Briefly, DNA k-mers of a predefined length are counted in each contig under analysis, and a clustering or classification algorithm is then run to group together the sequences with similar composition. The classification is in this case completely unsupervised, as it makes no use of available taxonomical information. This allows the identification of OTUs with no counterpart in public repositories, and consequently the resulting OTUs can only partially be mapped to existing taxonomic groups, depending on the availability of similar DNA sequences.

Currently, a large number of reference-free methods are available, as they are easily scalable to the size of present-day metagenomes; here, we briefly describe some recent implementation that we used to put in scale the performance of our tool. MetaBAT2 (Kang et al., 2019) uses tetranucleotide frequencies and coverage to calculate sequence distances and group them using a K-medoid clustering approach. The calculation of the distances can be unstable with short sequences (the authors suggest to avoid using contigs shorter than 2 kbp). MaxBIN (Wu et al., 2014) implements an expectation maximization algorithm where tetramer frequencies and coverage are used separately to calculate the probability that two contigs come from one and same genome; the probabilities are then combined, up to convergence of the parameters. The software provides additional information about the identified bins, like inferred genome sizes, GC content, completeness, and coverage. BusyBee Web (Laczny et al., 2017) bins sequences in metagenomic samples by using a hybrid classification approach: calculation of the k-mer frequencies (either 4 or 5 bp long) is followed by unsupervised binning on a subset of the data using DBSCAN (Ester et al., 1996); at the end, a random forest (RF) (Cutler et al., 2007) model trained on the labels from the unsupervised step is used to predict the unused part of the data. BusyBee can moreover integrate the identified bins with Prokaryotic taxonomic information or user-provided custom affiliations.

The above methods are based on higher-order compositional properties of the contigs, where the order refers to the length of the k-mers; the phylogenetic signal of DNA composition is related to differences in codon usage, mechanisms of DNA modification, replication, repair, and site recognition by endonucleases, thus leading to a highly species-specific information (Karlin et al., 1994; Karlin and Burge, 1995; Karlin et al., 1998; Gentles and Karlin, 2001). It has been shown that the resolution of composition-based phylogenetic methods improves when increasing the length of the k-mers, up to a limit that depends on the length of the sequences where the k-mers are counted. Intuitively, the longer the k-mer, the longer the DNA sequence has to be to minimize the noise in the counts. As a rule of thumb, we should restrict the counting to sequences that are at least 10 times the number of possible k-mers of a certain length; with 5-mers there are 1,024 possible words, and the minimum length of the contigs should be set at ∼10kb and therefore, depending on the assembly in hand, applying such stringent thresholds on the length of the contigs might reduce too much the information used.

Here we present SeqDex, a tool written in R that combines partial taxonomic affiliations obtained through homology searches combined from different databases with composition analysis to predict the taxonomic affiliations of all the contigs present in an assembly produced from the sequencing of mixed samples involving a host and its endosymbiont(s). SeqDex is innovative as it additionally implements a graph-based strategy to transfer taxonomical labels and because we provide a full characterization of the performances in a case-by-case way, helping the user to understand how effective is the classification. We provide both a comparison with similar methods and several performance measures to summarize and rank the different tools.

## Methods

### The SeqDex Workflow

SeqDex couples both unix-based programs and custom R script developed using the following R packages: Seqinr (Charif and Lobry, 2007), Taxonomizr (cran.r-project.org/web/packages/taxonomizr/index.html), randomForest (Liaw and Wiener, 2002), e1071 (cran.r-project.org/package = e1071), uwot (cran.r-project.org/package = uwot), dbscan (Ester et al., 1996), igraph (Csardi and Nepusz, 2006).

#### Coverage Calculation

SeqDex calculates sequencing depth using the BEDtools coverage (Quinlan and Hall, 2010); fragment counts per contig are then normalized by contig length. SeqDex considers the case of using FLASh or similar software to merge overlapping reads. Such software produces single-end reads when a pair was overlapped; otherwise, both reads in a pair are kept. When this happens, SeqDex considers the paired- and the single-end reads separately to provide a correct estimation of the coverage expressed in number of sequenced DNA fragments divided by the length of the contig. Moreover, when the mates map on different contigs, they contribute half a count.

#### k-mer Frequencies Calculation

GC content and k-mer frequencies are calculated by SeqDex with the Seqinr package on both strands. The counts for complementary k-mers are combined such that all k-mers, comprising the palindromes, get the same weight. As count matrices for long k-mers have a high dimensionality, the computational time needed for the analysis increases, and this step provides a reduction of the dimensions with no loss of information. SeqDex considers contigs longer than a user-defined threshold as short sequences can diverge from the genome composition reducing the prediction capability of the model.

SeqDex by default calculates frequencies for 3-mers on contigs longer than 1 kbp.

#### Taxonomy Affiliations

SeqDex assigns taxonomy affiliations to contigs on the basis of homologies: contigs are compared to a nucleotide database by using BLAST plus (Camacho et al., 2009), after filtering at a defined percentage of identity and length of the HSP (high scoring pair). Thresholds can be changed by the user by editing the SeqDex bash script (see below for default values). The association of taxonomical codes to contigs is obtained by using Taxonomizr. As contigs potentially have multiple homologs in the database, SeqDex calculates the proportion (TaxonDensity) of alignments pointing toward a certain taxonomic category over the whole set of homologies for each contig. All contigs with a TaxonDensity value below the defined threshold have their taxonomy label removed; in this way, SeqDex reduces the risk of wrong or inconsistent taxonomical assignments by removing contigs for which we get incongruent taxonomic affiliations. The database can be defined by the user, and it is used to assign a taxonomic origin to contigs in the input. The only limitation is that sequence titles must conform to NCBI format.

One of the problems with symbiont genomes is the low identity level with related genomes; we endowed SeqDex with the functionality of merging the taxonomic information retrieved for both nucleotide and proteins. Protein evolutionary rates are much slower than those characterizing nucleotide sequences, meaning that in some case it might be possible to find homologs by using the protein but not the corresponding gene. Protein coding genes are predicted with Prodigal (Hyatt et al., 2010) (default options, except for procedure set to “meta”), and translated sequences are then compared to a protein reference database by using Diamond (Buchfink et al., 2014) (default options). As before, the reference database may be the NCBI nr or a custom database with titles in NCBI format.

We stress the fact that considering the taxonomy coming from protein comparisons can be particularly advantageous when the symbiont is from taxa that are underrepresented in the public repositories. However, adding the protein derived taxonomy affiliations may increase computational time needed. In low complexity datasets, the use of protein affiliations may not increase the performance of SeqDex; thus, we decided to leave to the user the decision to use this information or not.

SeqDex exploits the presence of 16S genes within the assembly, to identify the contigs of the target organisms in a final step of the workflow. rRNA genes in the assembly are identified by using Barrnap (https://github.com/tseemann/barrnap), and then contigs with 16S genes are compared to RDP 16S database by using BLAST to add a taxonomic label to the 16S gene.

All homologies detected by BLAST and Diamond are reported, not only the best ones, allowing to compare multiple significant taxonomy affiliations for the same contig, which may highlight incongruencies.

If both nucleotide and protein homologies are used, SeqDex merges the affiliations and assigns a unique label to contigs.

By default, SeqDex considers contigs longer than 1 kbp, nucleotide (protein) HSP length over 200 bp (70 aa), percentage of identity over 70% (80%), and a final TaxonDensity larger than 0.75 (implying that 75% of the HSPs in the BLAST provide the same taxonomic information).

#### Extending Taxonomy Information

Symbionts have often evolved for long time in an environment endowed with a very peculiar fitness landscape; this translates in their usually divergent genomic properties (extremely reduced gene content, AT richness, etc.). Furthermore, we are only scratching at the surface of the diversity of existing symbionts, and very often the study of novel symbionts leads to the discovery of novel genera or even families (Castelli et al., 2019). For the above reasons, the taxonomy assignment based on blast generally leads to relatively few contigs being labeled. This affects the parameterization of the machine learning models in a negative way, as it reduces the number of labeled cases. One way to cope with this is by including protein comparisons; therefore, SeqDex also allows to integrate this kind of analysis. Nonetheless, the taxonomy coverage of contigs from symbiotic communities is often low (i.e., only a small fraction of contigs has significant similarities to sequences in the database).

To further improve the taxonomy coverage of a sample, SeqDex exploits the information related to the paired end reads mapped back on the assembly. Basically, SeqDex builds a graph where two vertices (contigs) *a* and *b* are connected if there is at least one pair of reads for which one mate maps on contig *a* and the other on contig *b*. This graph is related to what assemblers use for scaffolding. Edges are weighted by the number of read pairs in support; therefore, they can be filtered to only keep the highly supported ones, by defining a threshold in the SeqDex bash script (EDGES, default = 10). If we assume that the genomes present in the sequenced pool are different enough, as we expect in host–symbiont cases, then the connected components (CCs) in this graph mostly comprise vertices corresponding to regions from the same genome. Therefore, the taxonomy label associated to one vertex can in principle be transferred to vertices of the same CC. Reads can, however, randomly map on genomes from phylogenetically distant genomes, and filtering edges on their weight provides a way to remove most of the chimeric associations. Additionally, the user can control the maximum degree of vertices, as highly connected ones are more prone to be responsible for the connections involving contigs from different sources (VERTICES, default = 5). As a partial error control strategy, SeqDex checks for discordant taxonomical signals within each CC on the basis of the homology-defined taxonomy, and it only applies the transfer when most labeled contigs in the CC provide the same information (can be controlled by setting MIXEDCOMP, default = 0.2). Alternatively, the user can choose to transfer the labels up to a certain distance from labeled vertices (VERTEXDIST, default = all), which may be a compromise between the risk of wrongly propagating taxonomic information (likely reduction in precision when extending taxonomic information far away from labeled vertices) and the opposite risk of strongly reducing the recovered information (likely reduction in recall when not transferring labels to short contigs, especially in the case of highly fragmented assemblies). In this case, transfer proceeds up to the defined distance from the labeled node, but if node n has a different taxonomy label, the transfer is performed up to node n-2. The increment of performances related to this approach is shown in **Supplementary Table S7**.

#### Predicting Taxonomic Affiliations

The above steps prepare the input for the classification tasks performed by SeqDex and are given by the matrix containing k-mer frequencies for each contig and the corresponding taxonomic affiliations. In a standard run, SeqDex will perform a classification of the contigs first at the level of Superkingdom. In this way, host and symbiont/contaminants contigs are separated. Model training is performed with RandomForest and Support Vector Machine (SVM). In both cases, SeqDex performs model training 100 times on 66% of the contigs fulfilling all thresholds and calculates the performances of the classification on the remaining 33%. All models are kept in memory and are used for performing the classification of the contig without a taxonomy label. Different models can assign different labels to the same contig; therefore, after 100 predictions, SeqDex returns the percentage of times each contig was included in a certain taxonomy category, and the final label corresponds to the category with the highest percentage.

#### Extending the Predictions

In the next step, the graph obtained by exploiting the pairing information is used to improve the predictions at the end of the classification, through a transfer strategy and consistence check similar to the one used for extending taxonomy labels. The feasibility of such a transfer is decided on a case-by-case basis by following the same rules defined in section “Extending taxonomy information” with the difference that, when it is not possible to extend the predictions, the taxonomy labels predicted for contigs are discarded (and marked as ‘misclassified’) instead of being kept.

#### Unsupervised Clustering

In a hypothetical condition, only host and endosymbiont genome sequences will be present in the dataset, so the classification step at Superkingdom level will allow to retrieve the bacterial contigs. However, this is rarely the case. Usually, contaminants are also present, but we noticed that SVM and RF are not able to provide satisfactory performances in these cases (data not shown). For this reason, SeqDex performs a final step to cluster sequences in groups of similar composition and, thanks to the identification of the cluster containing the target 16S gene, is able to recover contigs deriving from the target genome. By enabling this optional analysis (CLUSTERING, default = yes), SeqDex will (i) take the output of the classification step at the lowest selected taxonomic rank, (ii) apply a UMAP transformation to the data (R package uwot, NCOMP, default = 2), (iii) cluster the new variables with DBscan (Ester et al., 1996), (iv) identify the cluster comprising the contig carrying the target 16S gene, and (v) flag all the contigs in the same cluster as belonging to the target genome. This is the final taxonomy prediction made by SeqDex, and the results are based on this set of contigs.

If several 16S genes with the taxonomic affiliation of interest are present, SeqDex will use the one with the highest coverage.

As discussed before, coverage and/or GC content may be also highly informative, depending on the specific symbiotic system. In these cases, the user can decide to perform the clustering by adding the coverage and/or the GC content to the data matrix storing the UMAP coordinates (TYPE, can be k-mers, gc, cov, and combinations thereof, e.g., TYPE = k-mers,gc adds the GC content as a variable in the clustering together with k-mers; default = k-mers).

#### Extending the Final Taxonomy Prediction

As done at the end of the machine learning classification, the clustering can be improved by using the same transfer strategy based on the read pairs-based graph. This is because methods based on k-mers are meaningful only when performed on contigs above a certain length (which depends on the selected k). Sometimes, a consistent proportion of the assembly is excluded for this reason, with information loss. However, since short contigs are present in the graph built using the pairing information, SeqDex transfers the clustering belonging within a CC as done in “Extending the prediction.”

#### Standard SeqDex Output

In standard usage, SeqDex produces the following output files:

Taxonomy folder: several files for the homology searches and taxonomy affiliations.
Coverage folder: k-mer frequencies, GC content, coverage;
SVMoutput and RFoutput folders: input and output files for the machine learning step.
ClusteringOutputSVM and ClusteringOutputRF folders: several files related to the DBscan clustering output. More specifically, this folder also contains the file with the name of the contigs in the target clusters and the fasta file with the sequences of the target contigs.

### Data

To develop and test SeqDex, we used three datasets: (i) a simulated dataset composed by *Saccharomyces cerevisiae* and *Neisseria gonorrhoeae*; (ii) a published dataset of an endosymbiont sequenced together with the host (*Ca. Fokinia solitaria*) (Floriano et al., 2018) that was extensively curated and resequenced to complete and close the genome; (iii) a dataset of a nematode (*Pratylenchus penetrans*) sequenced with his two endosymbionts (*Wolbachia pipientis* and *Ca. Cardinium hertigii*) (Brown et al., 2016; Brown et al., 2018); in this case, the genomes of the two symbionts were only partially assembled by the authors.

#### Simulated Dataset

We simulated paired-end reads from the *S. cerevisiae* and *N. gonorrhoeae* genomes by using the wgsim package (https://github.com/lh3/wgsim) changing the following parameters: number of read pairs set to one million; read length: 100 bp; fraction of indels set to 0.01; probability that an indel is extended equal to 0.05; outer distance between the two ends of 2000bp. Paired reads for both genomes were randomly sampled and merged to obtain a dataset composed by *S. cerevisiae* and *N. gonorrhoeae* in a 9:1 proportion. We assembled the reads using SPADes (Bankevich et al., 2012), with k-mer length ranging from 31 to 91. We selected the best assembly using QUAST (Gurevich et al., 2013) based upon the N50 statistic. SeqDex were run using only nucleotide comparisons against a custom database composed by the two genomes present in the dataset, classification at Superkingdom level with k-mers of length 3, and both machine learning algorithms, and the clustering was disabled.

#### Real World Dataset—*Ca*. F. Solitaria, Endosymbiont of a Ciliate

*Ca*. F. solitaria and its host were sequenced using Illumina HiSeq 2500 to generate 14,783,394 150 bp paired-end reads, as reported by the authors (Floriano et al., 2018). We assembled the reads obtained by the authors using SPADes, with k-mer length ranging from 31 to 91 and then chose the best assembly using QUAST, based on the N50 statistics. In SeqDex, taxonomies were assigned using BLAST against the NCBI nt database (downloaded in October 2018), with default options but excluding *Ca*. F. solitaria genome; the 16S rRNA genes were compared to RDP 16S database downloaded in October 2018. SeqDex was run with default parameters using *Alphaproteobacteria* as target class for the final clustering.

#### Real-World Dataset—*Wolbachia–Cardinium* Dual Endosymbionts of a Nematode

*P. penetrans* and its endosymbionts where sequenced using Illumina MiSeq to generate 301-bp-long paired-end reads (accession: SRR3097580) for a total of 10,563,810 pairs (Brown et al., 2016). Reads were quality checked with fastQC (Andrews, 2010), adapters were removed using Trimmomatic (Bolger et al., 2014), and overlapping pairs were merged using FLASh. Assembly was performed with SPADes, using default parameters and k-mer length of 21, 33, 55, 77, 99. The best assembly was chosen using QUAST. SeqDex was run on both endosymbionts using both nt and nr NCBI databases, the same RDP 16S databases of *Ca*. F. solitaria, two classification iterations on Superkingdom and class taxonomic levels, and the final clustering searching as target “unclassified_Bacteroidetes” for *Ca*. C. hertigii and “Alphaproteobacteria” for *Wolbachia*.

### Performance Calculation

Regarding the study cases shown in the article, we calculated additional statistics to highlight the behavior of SeqDex that exploit the availability of genome sequences of the symbionts as from previous publications (Brown et al., 2016; Brown et al., 2018; Floriano et al., 2018). In these cases, we perform comparisons of the performances of the different methods based on counting true positives and negatives (TP and TN, respectively), false positives and negatives (FP and FN, respectively), which are used to calculate sensitivity, accuracy, precision, and F1 scores (**Supplementary Table S1**). We stress that these statistics can be calculated here because the true labels can be derived for all contigs, thanks to the availability of the symbiont genomes. The performances of tools performing the taxonomical classification of contigs are generally based on numbers of correct/wrong classifications; however, the many contigs obtained from short reads have very heterogeneous lengths such that weighting the error made in the classification with respect to the length of the sequences should provide a much better characterization of the true capability of a tool. For instance, a tool misclassifying a very short contig performs better than one misclassifying a very long one, and yet both have the same performances if we refer to raw contig counts. For this reason, we also compare the contigs assigned to each taxonomical category with the source genome, and we calculate performances based on the total number of nucleotides that were correctly assigned, with respect to the genome length. To evaluate this, we used QUAST, which provides a comparison among an assembly and a reference genome.

### Details About Third-Party Tools Parameters

We compared SeqDex with the methods Blobology (Kumar et al., 2013), MetaBAT2 (Kang et al., 2019), BusyBee (Laczny et al., 2017), and MaxBin (Wu et al., 2014). If not otherwise specified, only contigs longer than 1,000 bp were considered, with GC content and coverage values calculated as described before.

MetaBAT was run on contigs by changing minimum contigs length (-m, set to >1,500 bp), percentage of “good” contigs (–maxP, set to 90), minimum score of an edge for binning (–minS, set to 80), and minimum size of a bin as the output (-s, set to 150,000). MaxBIN was performed using default parameters, and only k-mer length was changed in BusyBee Web (*k* = 4).

## Results

### SeqDex Pipeline

SeqDex is written mainly in R but can be run from a bash script where the user can change most parameters (**Supplementary Table S2** lists all scripts that are part of SeqDex, and that are available for download at https://github.com/ComparativeSystemsBiologyGroup/SeqDex).

**Figure 1:**
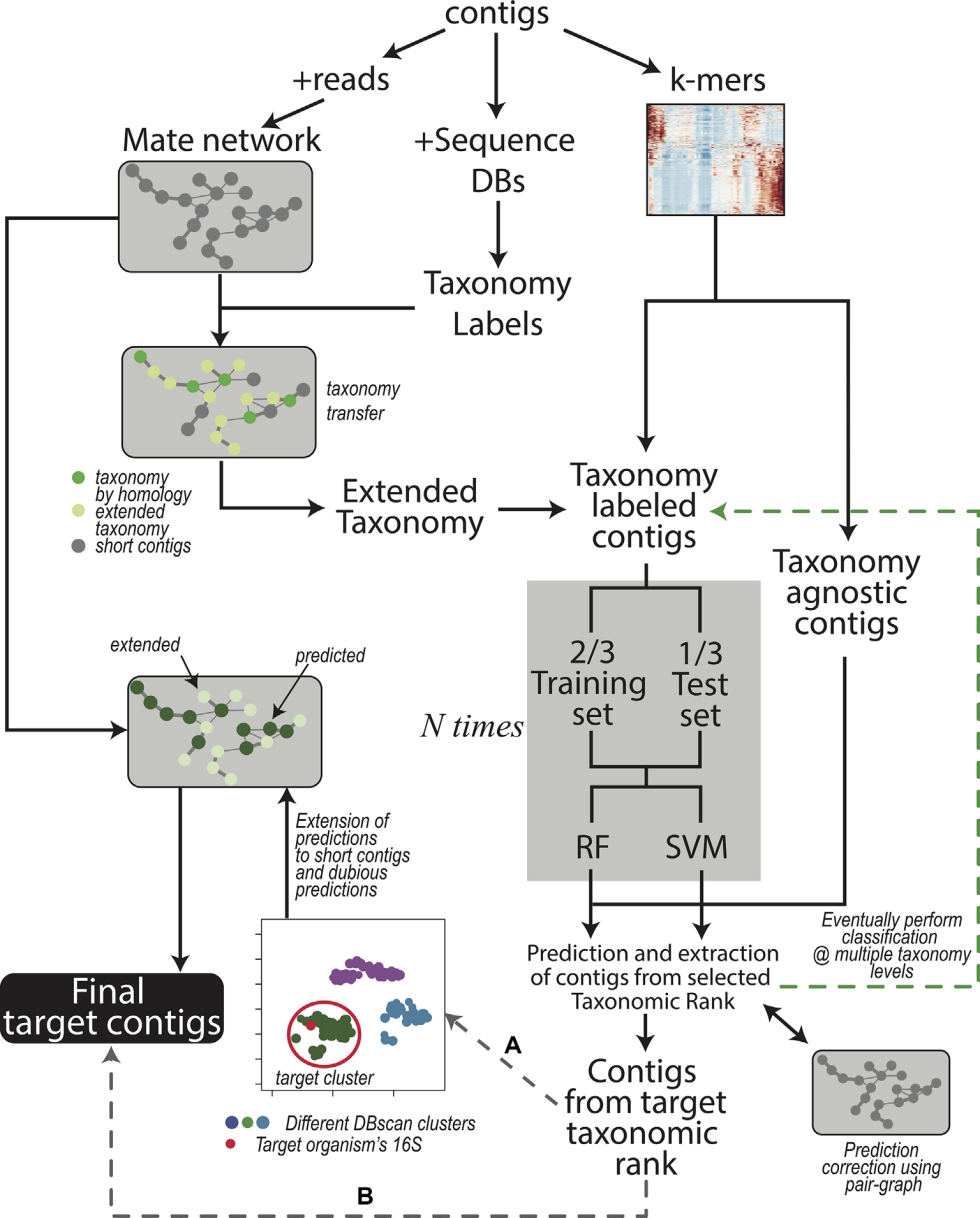
Contigs are used to obtain the read-pair graph by exploiting the paired sequencing (left branch). The network is used in several steps of the procedure, for instance, to extend the taxonomy information obtained through sequence comparison (middle branch). The k-mer frequencies are also calculated (right branch) and combined with the (extended) taxonomy. The contig dataset is then split in two depending on the presence of taxonomy labels; the labeled contigs are used to train the machine learning models (gray box) after partitioning the contigs again into a training and a test set. Training of the models is repeated N times to provide error estimations that are independent of the actual contigs in the train and test sets. As a default, classification is performed at the only Superkingdom level; if the user wants to proceeds down in the taxonomy hierarchy, additional iterations, each time focusing on a different taxonomic rank (green branch), can be performed. After that, SeqDex uses the trained models to predict the taxonomic affiliations of unlabeled contigs. Again, the read-pair network can be used to correct the predictions made by the machine learning models. At this point, contigs can be recovered, and two possible alternatives exist: **(A)** when there is more than one bacterium in the sequencing, the user can proceed by directing SeqDex on the flow indicated with **(A)**: (i) run UMAP, (ii) DBscan on the UMAP transformed k-mer frequencies, (iii) identify the cluster containing the target 16S gene, (iv) extend predicted taxonomy information using the read-pair graph, and (v) extract the contigs identified as coming from the target organism. Alternatively, **(B)** SeqDex can directly extract the contigs classified as coming from the target organism after the machine learning step.

The workflow is shown in **Figure 1** and each step is described in Methods:

1. Coverage calculation. We indicate the whole set of contigs in the assembly as A;
2. Identification of 16S rRNA genes to identify target bacteria (the identity of the symbiont in these situations is often achieved through polymerase chain reaction [PCR] amplification and sequencing);
3. Comparison with sequence databases to associate contigs to taxonomic affiliations for a subset T (with T ⊆ A) of the contigs; this can additionally be performed at the protein level;
4. Taxonomy extension using the paired read graph of the assembly;
5. k-mer frequencies are calculated for all contigs in C (C ⊆ A such that contigs in C are longer than a defined threshold).
6. Random forest (Cutler et al., 2007) and SVM (Cortes and Vapnik, 1995) models are trained on data for contigs in T∩C by exploiting k-mer frequencies and the partial taxonomical affiliations obtained in 3 and 4. Then, the trained models can be used to predict taxonomical affiliations of the contigs with no taxonomical label. At this point, all contigs in C have a taxonomical affiliation, coming from step 3 and 4 or predicted here. The problem is split into nested classifications by considering different taxonomical depths: a first classification separates prokaryotic from eukaryotic sequences; contigs included in the former can then be used for more stringent classifications by applying the model at a stricter level of taxonomical categories. At the end of this step SeqDex provides a taxonomy for the contigs. This can be used as is or it can be processed in the following optional step.
7. Useful when the user knows there may be more species in the assembly (e.g., bacterial contaminants, in addition to the target organism(s)). The machine learning approach has unsatisfying performances at this level (data not shown) and therefore was replaced by the following strategy. First, the k-mer matrix undergoes dimension reduction using the UMAP transformation (McInnes et al., 2018); then a DBscan clustering (Ester et al., 1996) provides a partition of the contigs into clusters. The cluster containing the 16S rDNA gene with the right taxonomical affiliations is defined as the target cluster. Then, paired-end reads mate graph is used to control the clustering and also to extend it to contigs shorter than the defined threshold (A–C), so that the final target cluster contains also contigs that were excluded in (5). The contigs falling in the target cluster are now retrieved.

The entire SeqDex procedure can be run using default parameters, but the scripts are customizable, as the user is able to change several key parameters.

### Case Studies

#### Simulated *S. cerevisiae–N. gonorrhoeae* Dataset

We use this simulated dataset as an example of a very simple case with a eukaryote (as the host) and a bacterium (playing the role of the symbiont). Such simple situations are very rare in real-world cases, where usually more prokaryotes can be found, most of which are usually not the symbionts. Therefore, in this case, we proceeded by only classifying at the Superkingdom level, which could be done when preliminary analyses (e.g., PCR amplification) show the presence of only one bacterial 16S rDNA.

To assess the performance of the Blobology approach, we selected the contigs included in the region defined by GC content ≥0.3 and coverage ≤0.05 fragments/nt on the basis of an enrichment of contigs with the target taxonomic affiliation in that region.

BusyBee crashed reporting an error after identifying one only cluster in the dataset. MetaBAT completed the analysis but still found only one bin. The results of these two tools are therefore not shown for this study case.

MaxBIN correctly identified two bins, one mainly composed by contigs with taxonomic affiliation bacteria, which was selected as target.

Finally, we performed SeqDex with RF and SVM focusing on the Superkingdom level. Taxonomic affiliations were obtained by comparison to a database composed only by the genomes of the two organisms used for this simulation; thus, this homology search was enough to classify all contigs. To use SeqDex, we randomly discarded 33% of these affiliations.

**Figure 2:**
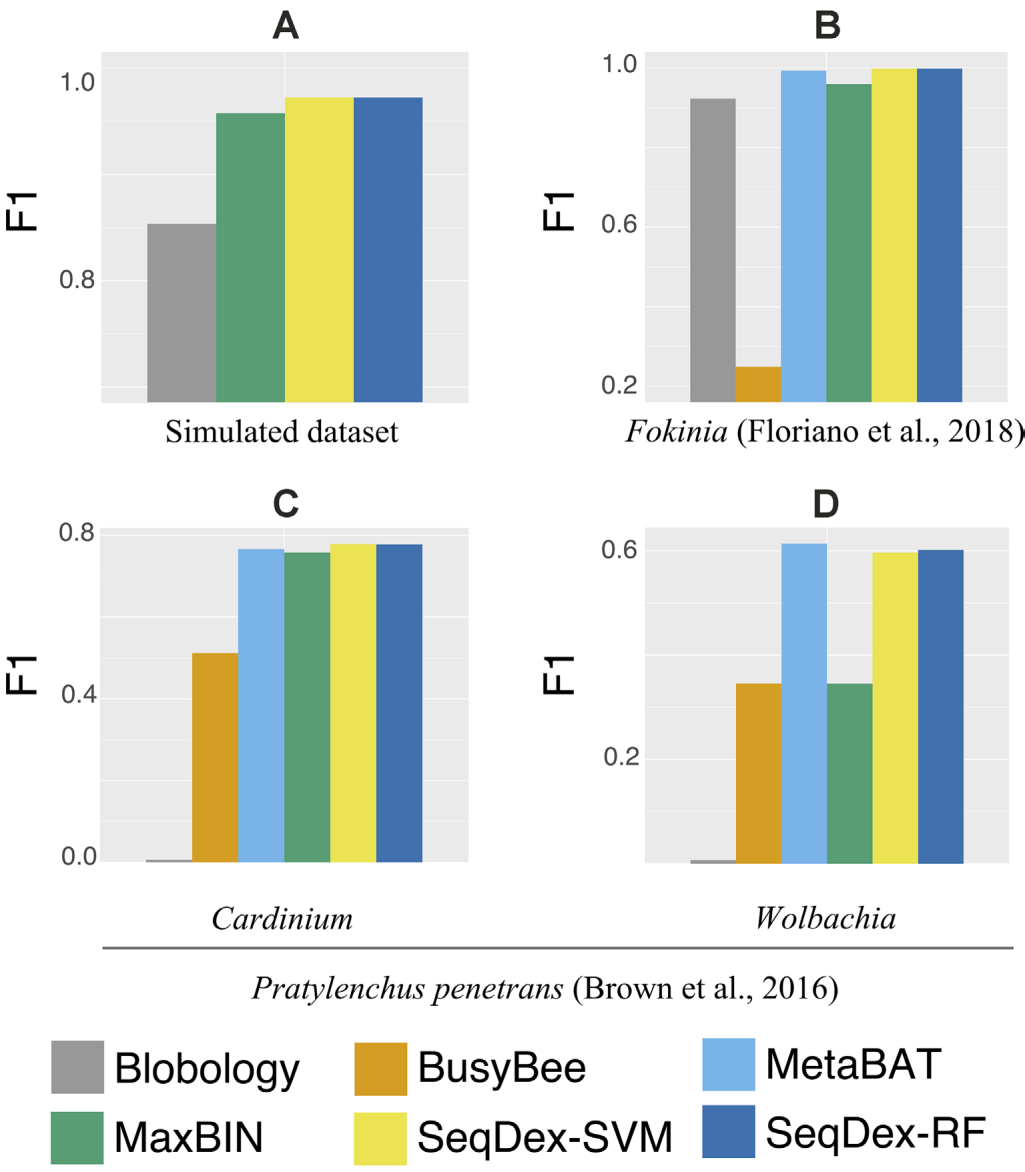
Genome-based F1 scores. For all datasets and targets considered in the work. **(A)** Simulated dataset; **(B)***Ca*. Fokinia solitaria dataset; **(C** and **D)***Pratylenchus penetrans* dataset: **(C)***Ca*. Cardinium hertigii; **(D)***Wolbachia pipientis*.

The contigs retrieved after each method were used to calculate the fraction of the genome of *Neisseria* and to calculate sensitivity, precision, accuracy, and F1 scores as described before (**Figure 2A** and **Supplementary Figure S1**, **Table 1** and **Supplementary Table S3**).

**Table 1:** Performances calculated with respect to the whole genome sequence of *Neisseriagonorrhoeae*.

	Blobology	MaxBIN	SeqDex-SVM	SeqDex-RF
Sensitivity	0.7442	**0.9634**	0.9586	0.9586
Precision	**1.0000**	0.9518	0.9862	0.9862
Accuracy	0.9909	0.9969	**0.9980**	**0.9980**
F1 score	0.8533	0.9576	**0.9722**	**0.9722**

Highest values in bold.

SeqDex outperforms Blobology in F1 score and sensitivity, both considering the total length and the number of contigs, but the latter has higher precision. This could be explained considering that these two organisms have different GC content and that the simulated sequencing produced widely different average coverages for the two genomes. Considering MaxBIN, SeqDex gives similar sensitivity and accuracy but higher precision and F1 score.

#### Real World *Ca*. F. solitaria Dataset

We applied the Blobology approach by using GC and coverage boundaries comparable to those used by the authors in the original publication (>30% GC, coverage between 0.3 and 8 fragments per nucleotide).

MetaBAT identified 10 bins, MaxBIN 5 bins and BusyBee Web 8 bins.

Our model exploited the presence of five complete 16S genes, belonging to class Gammaproteobacteria (RDP code: S000653219), Alphaproteobacteria (two different 16S genes with RDP codes S000607898 and S004400661, the latter of which 100% identical to *Ca*. F. solitaria), Epsilonproteobacteria (RDP code: S003597162), and Bacteroidia (RDP code: S001493056). Of these, the contig containing the 16S gene from *Ca*. Fokinia have a coverage of 2.78 fragments per nucleotide, while the others range from 0.02 to 0.27, reflecting the presence of a much higher copy number for the endosymbiont with respect to the other bacterial species in the sample.

For this dataset, we run the entire classification pipeline implemented in SeqDex: SVM and RF are used to define the contigs coming from eukaryotes and bacteria; then, the k-mer frequencies of the contigs with Bacteria affiliation (predicted or deriving from the blast), undergo the UMAP transformation that produces two new variables that DBscan uses to define clusters. The whole procedure resulted in 16 and 8 clusters for SVM and RF, respectively. In **Figure 2B** and **Table 2** we report the statistics relative to the cumulative length correctly classified by each approach, while performance statistics based on contig counts are shown in **Supplementary Table S4** and **Supplementary Figure S2**. Considering cumulative length, BusyBee web performed poorly: even if it has sensitivity values that are comparable to the other methods, its precision, accuracy and F1 score are extremely low. Blobology shows performance statistics comparable to MetaBAT, MaxBIN and SeqDex. It has to be considered that this represents an uncommon situation: host and endosymbiont have different GC content and the symbiont is very abundant, at least compared to other bacteria present, as the sample to be sequenced was carefully selected in lab on the basis of the strength of the 16S signal by *Fokinia*. Among the remaining tools, they all performed good, with MaxBIN having lower precision and F1 scores and MetaBAT shows lower sensitivity and F1 score than SeqDex. Among all, SeqDex with both machine learning algorithm shows higher sensitivity, precision, accuracy and F1 scores.

**Table 2:** Performances of the classifications with respect to the whole *Ca*. Fokinia solitaria genome.

	Blobology	BusyBee	MetaBAT	MaxBin	SeqDex-SVM	SeqDex-RF
Sensitivity	0.8684	**0.9966**	0.9864	**0.9966**	**0.9966**	**0.9966**
Precision	0.9843	0.1422	1.0000	0.9246	**1.0000**	**1.0000**
Accuracy	0.9973	0.8881	0.9997	0.9984	**0.9999**	**0.9999**
F1 score	0.9227	0.2489	0.9932	0.9592	**0.9983**	**0.9983**

Highest values in bold.

#### Real-World *Wolbachia–Cardinium* Dataset

This study case has additional levels of complexity because the host is multicellular; it contains at least two endosymbionts whose genomes are still incomplete. Moreover, as often for endosymbionts, the most closely related genomes from databases are not highly similar.

**Figure 3:**
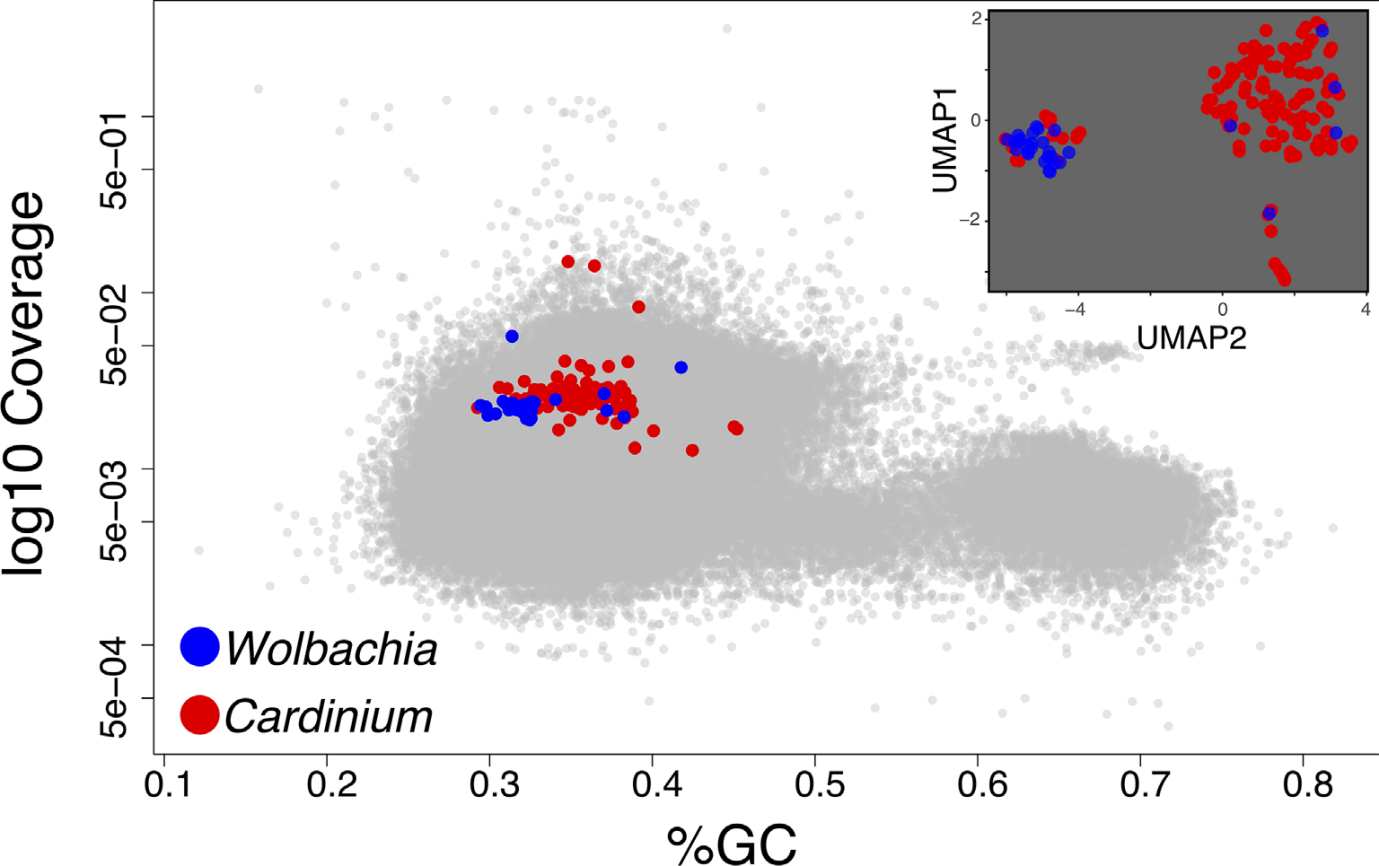
In the main panel, we show the Blobology plot obtained for the *Pratylenchus* dataset as an example of cases when host and symbiont(s) are not clearly discernible in the GC and coverage dimensions. In the inset, we represent the contigs from the symbionts (as identified through homology) in the UMAP space used by SeqDex to partition the contigs from the symbionts.

When plotting the contigs in Blobology space (GC Vs coverage), the sequences from the two symbionts did not form discernible clusters and also overlap with host’s contigs (**Figure 3**); it is therefore difficult to define regions enriched in sequences coming from one or the other symbiont and with the exclusion of host’s contigs. For the Blobology strategy, we tentatively defined the *Cardinium* region as defined by a GC content below 50% and by a coverage in between 0.001 and 0.3 fragments per nucleotide; the *Wolbachia* region was defined by a GC content below 40% and a coverage in between 0.01 and 0.1 fragments per nucleotide. We defined these thresholds based on the shape of the Blobology plot, by observing the location of contigs containing the symbionts 16S genes and exploiting the position of contigs aligning to the draft genomes available for the targets.

BusyBee identified 29 bins, but the two targets belong to the same bin. MetaBAT discovers 11 bins and MaxBIN 24, and both manage to assign one bin per symbiont. Our analysis exploited the presence of four complete and three partial 16S genes, belonging to Class Alphaproteobacteria (three complete genes with RDP codes: S003299234, S000830683, S001548999; the latter is identical to reference *W. pipientis* 99.98%), Gammaproteobacteria (a complete gene with RDP code S000711119), Betaproteobacteria (a partial gene with RDP code S000691097), and Cytophagia (a complete gene with RDP code S004482339 and a partial gene with RDP code S004414660, identical to *Ca*. *C. hertigii* at 100%).

In this dataset, we run SeqDex with two nested iterations. In detail, (1) taxonomic affiliations were used to predict the Superkingdom of contigs having no homologs in the database, and then we only select contigs predicted as bacteria (predicted and derived from homology); (2) the second iteration works on these contigs to predict the class; (3) contigs with Cytophagia affiliation were selected as potentially containing *Cardinium* contigs, and the additional clustering step was performed; (4) similarly, the contigs predicted as coming from the Alphaproteobacteria class were used for the clustering step to identify the *Wolbachia* contigs. SeqDex with SVM (RF) identified 8 (5) clusters within the Cytophagia dataset and 17 (6) within the Alphaproteobacteria one. As for the *Ca*. Fokinia, we then identified the cluster of interest by looking for the *Cardinium* and *Wolbachia* 16S genes. The performance statistics are summarized in **Figure 2C**, **Table 3**, **Supplementary Figure S3**, and **Supplementary Table S5** for the *Cardinium*; and **Figure 2D**, **Table 4**, **Supplementary Figure S4**, **Supplementary Table S6** for the *Wolbachia*.

**Table 3:** Performances of the classifications with respect to the whole *Ca*. Cardinium hertigii genome retrieved from the *Pratylenchus penetrans* dataset.

	Blobology	BusyBee	MetaBAT	MaxBin	SeqDex-SVM	SeqDex-RF
Sensitivity	**1.0000**	0.9781	0.8793	0.9289	0.9388	0.929
Precision	0.0033	0.3466	**0.6789**	0.6394	0.6649	0.6682
Accuracy	0.1423	0.9946	0.9981	0.9983	**0.9985**	**0.9985**
F1 score	0.0067	0.5118	0.7662	0.7575	**0.7785**	0.7773

Highest values in bold.

We concluded that the Blobology approach shows high sensitivity, comparable to other methods, but low precision, accuracy, and F1 scores for both organisms. BusyBee performed similarly to Blobology, except for accuracy, which is higher. Both methods basically failed to classify the two endosymbionts. Considering the total length of contigs correctly deconvolved for *Cardinium*, SeqDex, MaxBin, and MetaBAT showed comparable performance statistics, even though MetaBAT performed worse than the other two in accuracy, and SeqDex slightly better in F1 score. Instead, considering total length of *Wolbachia*, MaxBin performed worse than others in precision and F1 scores.

## Discussion

The comparison of our model with Blobology, BusyBee Web, MaxBin, and MetaBAT points out the generally superior performances of our method for all tested datasets.

In the simulated dataset Blobology, MetaBAT, and BusyBee Web failed to separate *Neisseria* from *Saccharomyces*, while SeqDex and MaxBIN showed similar good performances. In the *Ca. Fokinia* dataset, SeqDex and MetaBAT showed similarly good performance, while Blobology and BusyBee performed worse. In the *P. penetrans* dataset, SeqDex with both SVM and RF performed slightly better than MetaBAT and MaxBIN concerning *Cardinium*, and it outperforms MaxBIN on *Wolbachia*. By comparing the whole-genome–based performances for *Ca. F. solitaria* (**Figure 2B**, **Table 2**, **Supplementary Figure S2**, **Supplementary Table S4**) with those calculated for *Cardinium* (**Figure 2C**, **Table 3**, **Supplementary Figure S3**, **Supplementary Table S5**) and for *Wolbachia* (**Figure 2D**, **Table 4**, **Supplementary Figure S4**, **Supplementary Table S6**), we see they are lower in the latter. The published target genomes are, however, incomplete, and this might explain this difference, as the presence of correctly assigned contigs that are missing from the assembly would artifactually degrade the performances. Indeed, this dataset illustrates that SeqDex can also be helpful with complex datasets.

**Table 4:** Performances of the classifications with respect to the whole *Wolbachia pipientis* genome retrieved from the *Pratylenchus penetrans* dataset.

	Blobology	BusyBee	MetaBAT	MaxBin	SeqDex-SVM	SeqDex-RF
Sensitivity	0.9974	0.9922	0.9800	0.9810	0.9868	0.9868
Precision	0.0034	0.2091	**0.4469**	0.2096	0.4273	0.4327
Accuracy	0.5042	0.9936	0.9974	0.9936	0.9977	**0.9978**
F1 score	0.0068	0.3454	**0.6139**	0.3453	0.5963	0.6016

Highest values in bold.

We tested our model in a variety of condition: an unrealistic simulated dataset composed by only two organisms; a real dataset sequenced with high coverage, with a strong signal from the endosymbiont, and lower for nontarget bacteria; and a final real dataset containing two different endosymbionts and contaminant sequences. The performance analysis pointed out that SeqDex has comparable and sometimes superior performance to the other tools, which likely reflect the slightly different purpose for which most of the other tested tools were designed. The use of the paired-reads derived graph provides a boost to the performances when taxonomy labels derived from homology searches are particularly deficient. For instance, in all tested cases, the use of the graph to refine and extend taxonomies and predictions only provided a marginal improvement, except in the case of *Wolbachia* from the *Pratylenchus* dataset, for which the precision increases 10 to 100 times depending on the algorithm used for the classification. This shows that our procedure can be extremely helpful in particular cases.

In conclusion, SeqDex showed high reliability on all datasets, with high precision, accuracy, and F1 score. Differently from other tools, it provides and returns error estimation of the classification, such that the user understands if additional refinements are necessary and more importantly if the method can be applied.

We stress that in many situations it should be better to combine the output of different tools to achieve optimal results. This can be done in a conservative way, for instance, retaining only the contigs predicted as coming from the target by all applied tools, or using some sort of majority rule.

Future developments will focus on a modification of the machine learning algorithms to include sequence length-dependent weights for contigs (Freitas et al., 2007) as the machine learning algorithms that are commonly employed in these situations seek an optimization of the classification based on contig counts only (e.g., same weight is given to the wrong/right classification of a contig of 100,000 nucleotides and a contig of 2,000).

To conclude, another way to improve these approaches is the integration of clade-specific gene syntenies to further refine the composition-based classification.

## Data Availability

Publicly available datasets were analyzed in this study. This data can be found here: SRR3097580.

## Author Contributions

DS and GP defined the problem, and MB, CB, and DS developed the initial strategy and algorithm. AC developed all the codes and tested SeqDex and the additional tools under the supervision of MB. All the authors participated in manuscript preparation.

## Funding

This work was supported by Human Frontiers Science Program grant RGY0075-2017 to DS.

## Conflict of Interest Statement

The authors declare that the research was conducted in the absence of any commercial or financial relationships that could be construed as a potential conflict of interest.
