# Supplementary material for: SeqDeχ: A Sequence Deconvolution Tool for Genome Separation of Endosymbionts From Mixed Sequencing Samples
Source: Front Genet. 2019 Sep 19;10:853. doi: 10.3389/fgene.2019.00853 (PMC6761303; doi:10.3389/fgene.2019.00853)
Supplement: Supplementary file 2 [file Image_1.pdf]

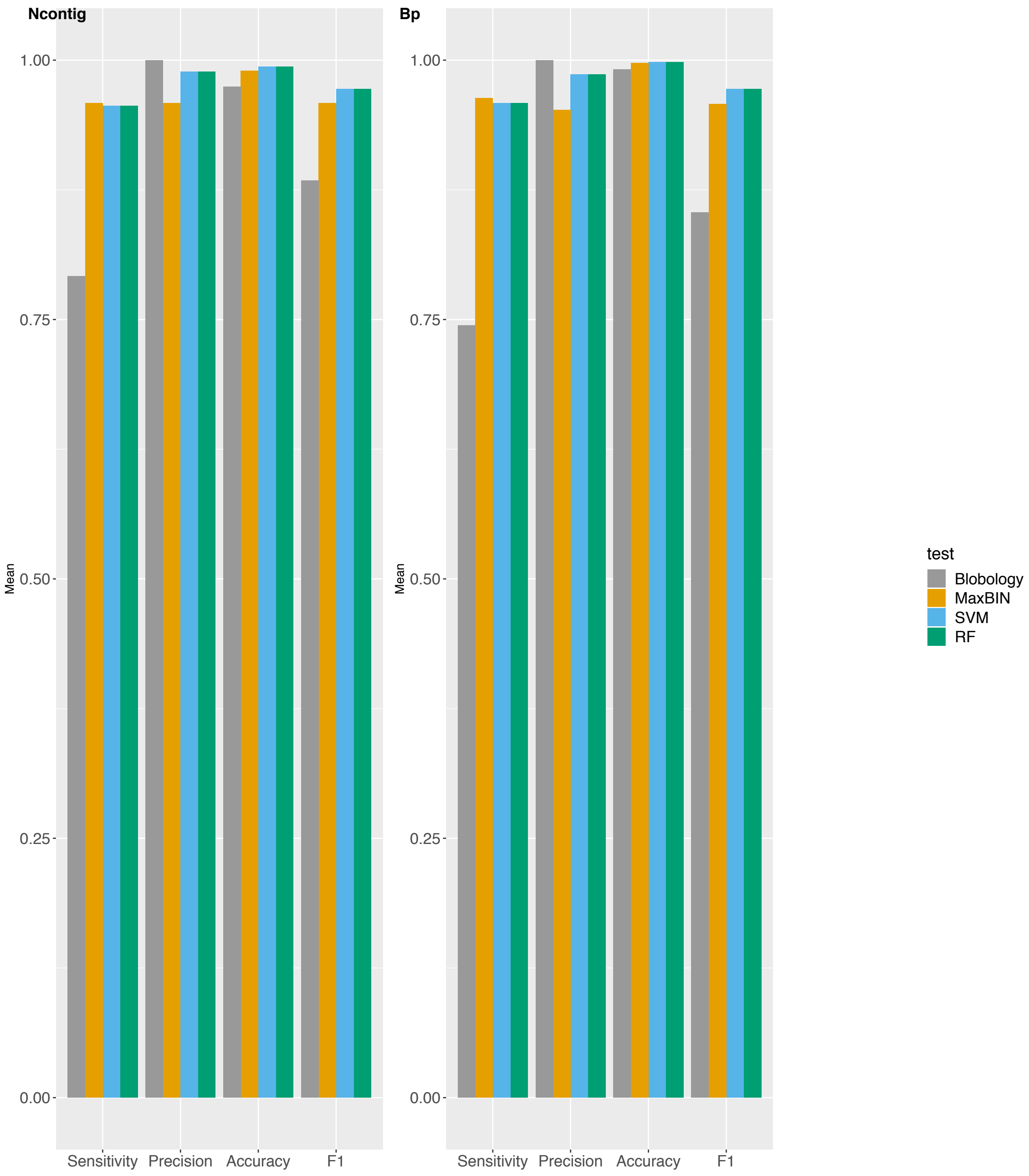

Supplemetary Figure S1 - The barplots show sensitivity, accuracy, precision and F1 score calculated for number of contigs (Ncontig) and amount of bp (Bp) of target organisms obtained by using Blobology, MaxBIN and SeqDex, with both algorithms, to deconvolve the simulated dataset.

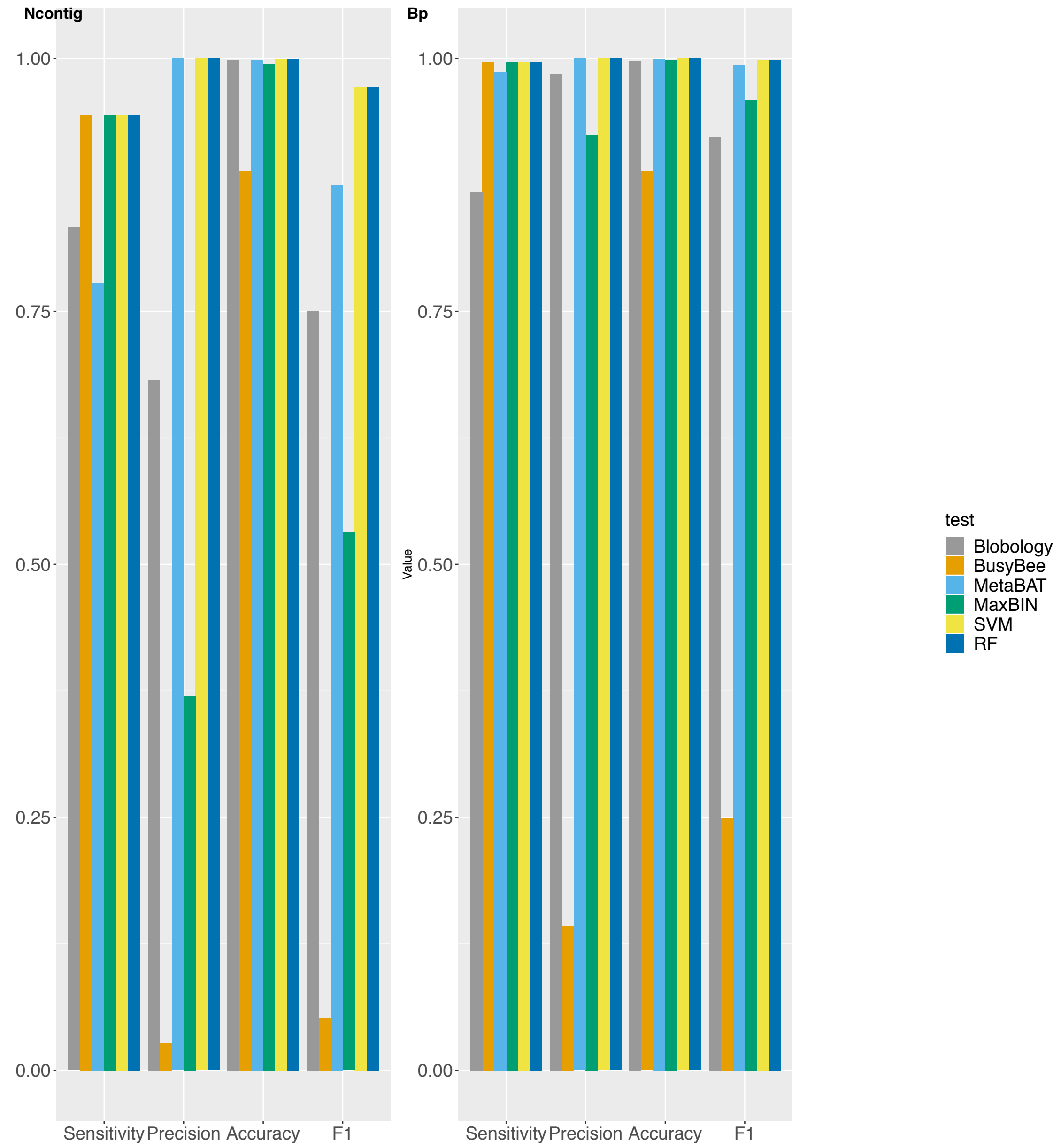

Supplementary Figure S2 - The barplots show sensitivity, accuracy, precision and F1 score of number of contigs (Ncontig) and amount of bp (Bp) of the *Ca*. Fokinia solitaria identified by using Blobology, BusyBee Web, MetaBAT, MaxBIN and SeqDex, with both machine learning algorithms, to deconvolve the dataset.

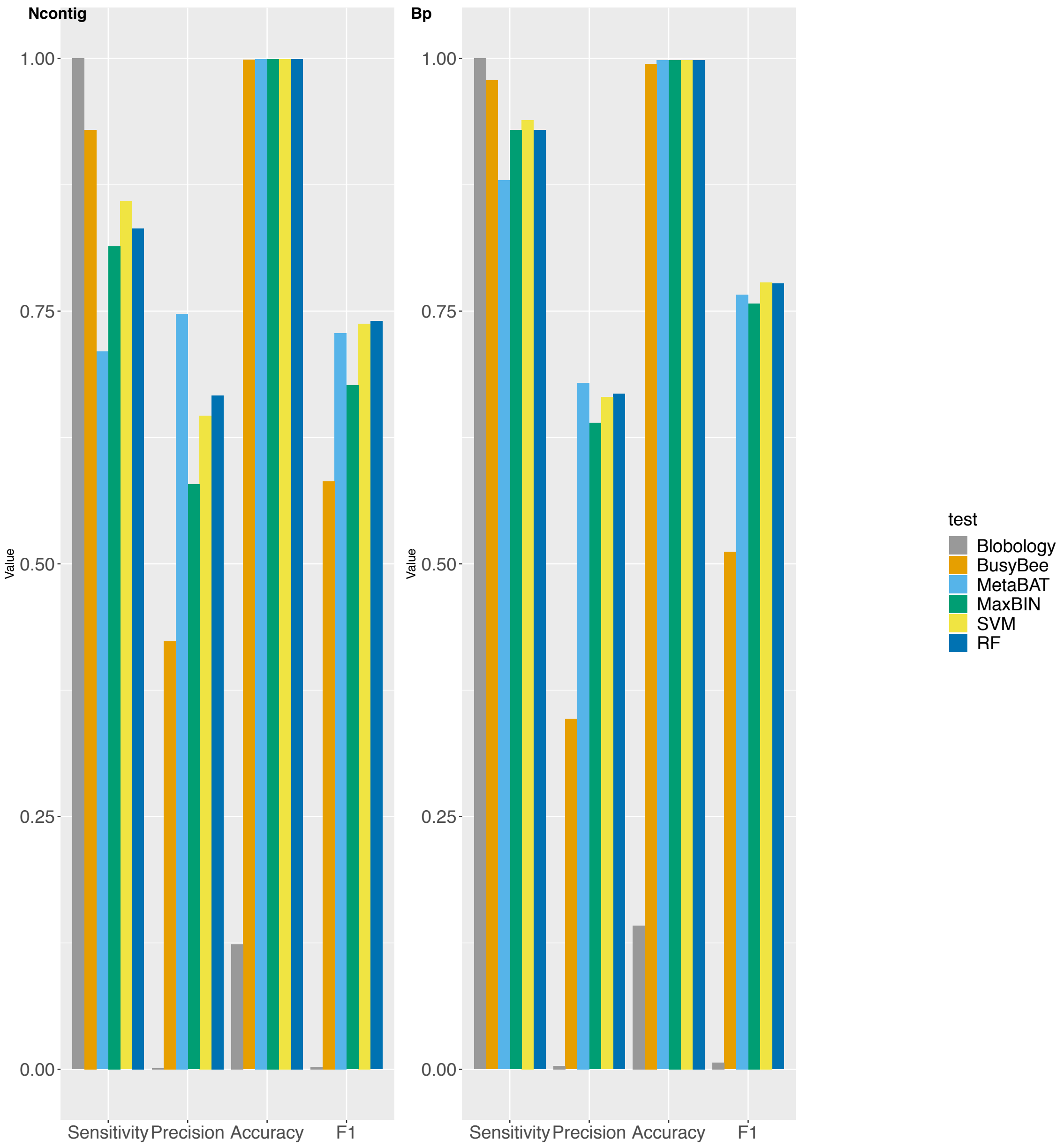

Supplementary Figure S3 - The barplots show sensitivity, accuracy, precision and F1 score calculated for number of contigs (Ncontig) and amount of bp (Bp) of *Cardinium* correctly deconvolved by using Blobology, BusyBee Web, MetaBAT, MaxBIN and SeqDex, with both machine learning algorithms, to deconvolve the dataset.

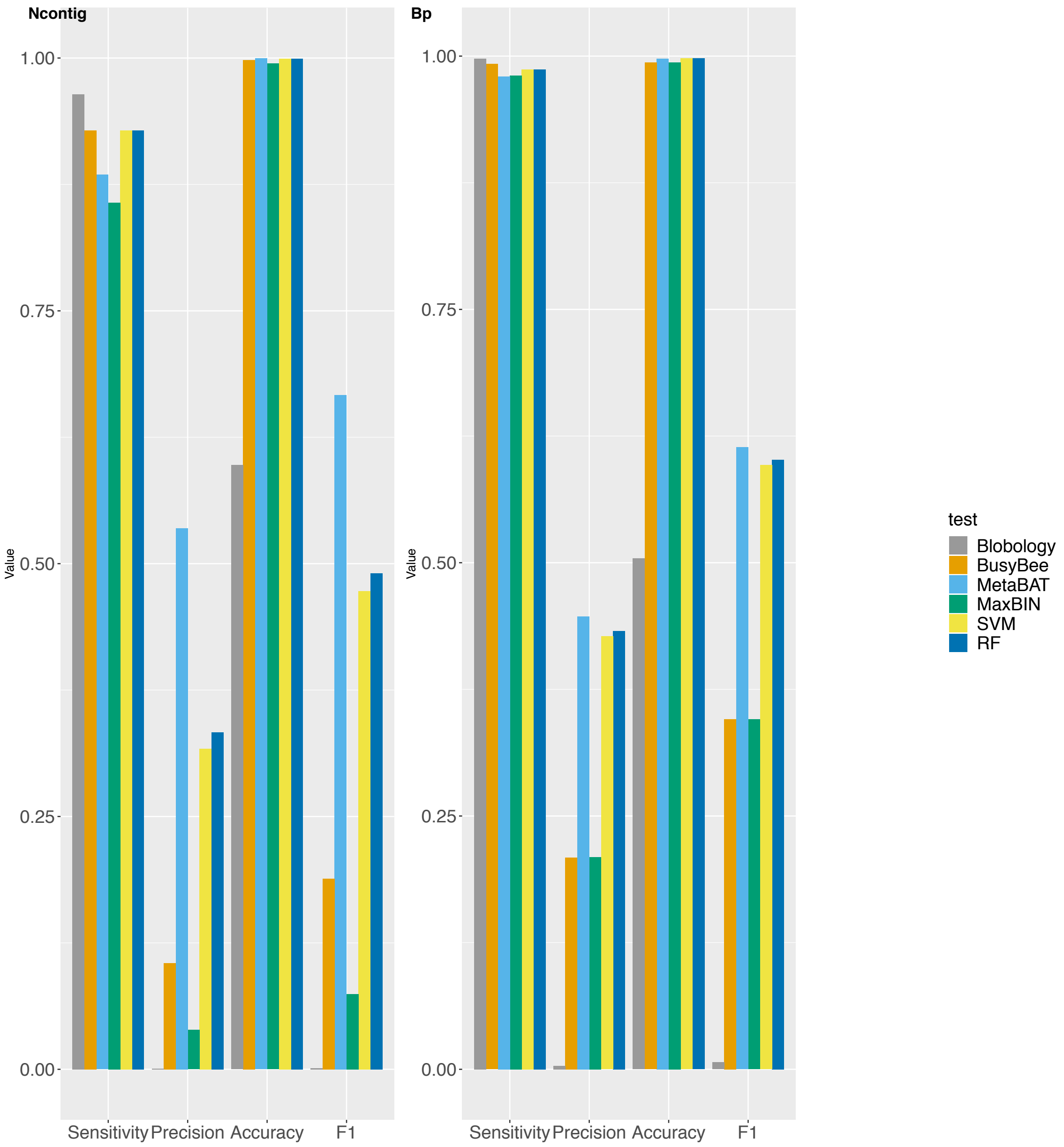

Supplementary Figure S4 - The barplots show sensitivity, accuracy, precision and F1 score calculated for number of contigs (Ncontig) and amount of bp (Bp) of *Wolbachia* correctly deconvolved by using Blobology, BusyBee Web, MetaBAT, MaxBIN and SeqDex, with both machine learning algorithms, to deconvolve the dataset.
